# Extended spectrum beta-lactamase and metallo beta-lactamase production among *Escherichia coli* and *Klebsiella pneumoniae* isolated from different clinical samples in a tertiary care hospital in Kathmandu, Nepal

**DOI:** 10.1186/s12941-017-0236-7

**Published:** 2017-09-19

**Authors:** Krishus Nepal, Narayan Dutt Pant, Bibhusan Neupane, Ankit Belbase, Rikesh Baidhya, Ram Krishna Shrestha, Binod Lekhak, Dwij Raj Bhatta, Bharat Jha

**Affiliations:** 1 Department of Microbiology, GoldenGate International College, Battisputali, Kathmandu, Nepal; 2 Department of Microbiology, Grande International Hospital, Dhapasi, Kathmandu, Nepal; 3 Department of Laboratory Medicine, OM Hospital and Research Center, Chabahil, Kathmandu, Nepal; 4 Central Department of Microbiology, Tribhuvan University, Kirtipur, Kathmandu, Nepal

**Keywords:** *E. coli*, *K. pneumoniae*, ESBL, MBL

## Abstract

**Background:**

Extended spectrum beta-lactamase (ESBL) and metallo beta-lactamase (MBL) production in *Klebsiella pneumoniae* and *Escherichia coli* are the commonest modes of drug resistance among these commonly isolated bacteria from clinical specimens. So the main purpose of our study was to determine the burden of ESBL and MBL production in *E. coli* and *K. pneumoniae* isolated from clinical samples. Further, the antimicrobial susceptibility patterns of *E. coli* and *K. pneumoniae* were also determined.

**Methods:**

A cross-sectional study was conducted at Om Hospital and Research Centre, Kathmandu, Nepal by using the *E. coli* and *K. pneumoniae* isolated from different clinical samples (urine, pus, body fluids, sputum, blood) from May 2015 to December 2015. Antimicrobial susceptibility testing was performed by Kirby-Bauer disc diffusion technique. Extended spectrum beta-lactamase production was detected by combined disc method using ceftazidime and ceftazidime/clavulanic acid discs and cefotaxime and cefotaxime/clavulanic acid discs. Similarly, metallo beta-lactamase production was detected by combined disc assay using imipenem and imipenem/ethylenediaminetetracetate discs. Bacteria showing resistance to at least three different classes of antibiotics were considered multidrug resistant (MDR).

**Results:**

Of total 1568 different clinical samples processed, 268 (17.1%) samples were culture positive. Among which, *E. coli* and *K. pneumoniae* were isolated from 138 (51.5%) and 39 (14.6%) samples respectively. Of the total isolates 61 (34.5%) were ESBL producers and 7 (4%) isolates were found to be MBL producers. High rates of ESBL production (35.9%) was noted among the clinical isolates from outpatients, however no MBL producing strains were isolated from outpatients. Among 138 *E. coli* and 39 *K. pneumoniae*, 73 (52.9%) *E. coli* and 23 (59%) *K. pneumoniae* were multidrug resistant. The lowest rates of resistance was seen toward imipenem followed by piperacillin/tazobactam, amikacin and cefoperazone/sulbactam.

**Conclusions:**

High rate of ESBL production was found in the *E. coli* and *K. pneumoniae* isolated from outpatients suggesting the dissemination of ESBL producing isolates in community. This is very serious issue and can’t be neglected. Regular monitoring of rates of ESBL and MBL production along with multidrug resistance among clinical isolates is very necessary.

## Background

The drug resistance among the gram negative bacteria is present as a serious global problem [1]. ESBLs are the important members of beta-lactamases produced mainly by gram negative bacteria [2] and are responsible for mediating resistance to extended-spectrum cephalosporins and monobactam aztreonam [3]. These enzymes are commonly detected in the members of the enterobacteriaceae like *Klebsiella pneumoniae* and *Escherichia coli* [3]. ESBL producing bacteria do not show resistance only to penicillins, most cephalosporins and aztreonam but also to other classes of antibiotics such as aminoglycosides, cotrimoxazole, tetracycline and fluoroquinolones [4, 5]. Further, the easy transmission of the ESBLs coding plasmids between the species has become a major threat mainly in hospitalized patients, often the infections caused by organisms producing ESBL being involved in outbreaks [6, 7]. Carbapenems are the drugs of choice for the treatment of the infections caused by ESBL producing bacteria [8]. However over the past few years, carbapenem resistance due to metallo-beta-lactamases (MBLs) production has been increasingly reported among clinical isolates from all around the world [9]. Metallo-beta-lactamase needs bivalent metal ions mainly zinc for its activation and resistance to carbapenems is mainly mediated by this enzyme [10].

The rapid increasing rate of MBL production among the members of enterobacteriaceae, mainly *E. coli* and *K. pneumoniae*, which are the most common causes of infections among human is present as a serious global public health problem [9]. There are limited treatment options for the infections caused by ESBL and MBL producing bacteria [11] due to which the treatments of such infections are very difficult often resulting into treatment failure. The regular surveillance of the drug resistance among the clinical isolates will be helpful to know the actual gravity of the situation, hence to formulate the necessary policy to reduce the incidence of drug resistance among the bacteria. Further, the knowledge about the local antimicrobial susceptibility patterns will be helpful to start timely proper preliminary treatment. So, in this study we determined the burden of ESBL and MBL production in *E. coli* and *K. pneumoniae* isolated from clinical samples. In addition, we also determined their antimicrobial susceptibility patterns.

## Methods

A cross-sectional study was conducted at Om Hospital and Research Centre, Kathmandu, Nepal, a 150 bedded hospital by using the *E. coli* and *K. pneumoniae* isolated from total of 1568 different clinical samples (urine, pus, body fluids, sputum, blood) from May 2015 to December 2015. The colonies grown after culturing of samples using standard microbiological techniques were identified with the help of biochemical tests [12, 13]. Antimicrobial susceptibility testing was performed by Kirby-Bauer disc diffusion technique following clinical and laboratory standards institute guidelines [14]. For performing antimicrobial susceptibility testing 90 mm diameter petri-plates were used and in one plate 6 antibiotic discs were tested. The diameter of the susceptibility zone was measured with the help of Vernier caliper.

### Screening and confirmation of ESBL producers

The isolates were screened for possible ESBL production using ceftazidime (30 μg) and cefotaxime (30 μg). According to the CLSI guidelines, the isolates showing reduced susceptibility to at least one of these drugs with zone of inhibition for ceftazidime ≤ 22 mm and cefotaxime ≤ 27 mm were considered as the possible ESBL producing strains. The suspected ESBL producing strains were confirmed for ESBL production by combined disc assay using ceftazidime (30 μg) and ceftazidime/clavulanic acid (30/10 μg) discs and cefotaxime (30 μg) and cefotaxime/clavulanic acid discs (30/10 μg). The zones of inhibition for the ceftazidime and cefotaxime discs were compared to those of the ceftazidime/clavulanic acid and cefotaxime/clavulanic acid discs. An increase in zone diameter of ≥ 5 mm in the presence of clavulanic acid was confirmed as positive for ESBL production [14]. Bacteria showing resistance to at least three different classes of antibiotics were considered multidrug resistant [15].

### Detection of MBL producers

The isolates showing resistance to imipenem were subjected to confirmation for MBL production by combined disc assay using imipenem and imipenem/ethylenediaminetetracetate discs [16].

## Results

Of total 1568 different clinical samples processed, 268 (17.1%) samples showed bacterial growth. Among which, *E. coli* and *K. pneumoniae* were isolated from 138 (51.5%) and 39 (14.6%) samples respectively. Out of 138 *E. coli*, 84% isolates were from urine followed by pus (7.9%). Likewise, out of 39 *K. pneumoniae*, 51.3% isolates were from urine followed by sputum (17.9%) (Table 1).

**Table 1:** Overall growth and distribution of *E. coli* and *K. pneumoniae* within different clinical samples

Samples	Total (%)	Bacterial isolates	
		*E. coli* (%)	*K. pneumoniae* (%)
Urine	184 (68.6)	116 (84.0)	20 (51.3)
Pus	22 (8.2)	11 (8)	2 (5.1)
Sputum	12 (4.5)	2 (1.4)	7 (17.9)
Catheter tips	7 (2.6)	2 (1.4)	5 (12.8)
Blood	20 (7.5)	3 (2.2)	3 (7.7)
Wound swab	14 (5.2)	4 (2.9)	0 (0)
ET suction tips	9 (3.4)	0 (0)	2 (5.1)
Total	268	138	39

### Distribution of the isolates on the basis of type and department of the patients

A total of 128 (72.3%) isolates were from outpatients and 49 (27.7%) from inpatients. Among the isolates, 78.3% and 21.7% of *E. coli* were obtained from outpatients and inpatients respectively. Similarly, 51.3 and 48.7% of *K. pneumoniae* were obtained from outpatients and inpatients respectively. Highest percentage of the isolates were obtained from general medicine department (35.5%) followed by obstetrics and gynecology (31.1%). Maximum percentage (35.5%) of *E. coli* were isolated from general medicine followed by obstetrics and gynecology (33.3%). Most of the *K. pneumoniae* were isolated from intensive care unit (25.6%) (Table 2).

**Table 2:** Distribution of the isolates on the basis of type and department of the patients

Patient types	Departments	Bacterial isolates (N = 177)		Total (%)
		*E. coli* (%)	*K. pneumoniae* (%)	
Outpatient	Emergency	7 (5.1)	1 (2.6)	8 (4.5)
	Obstetrics and gynecology	46 (33.3)	9 (23.1)	55 (31.1)
	Pediatrics	6 (4.3)	2 (5.1)	8 (4.5)
	General medicine	49 (35.5)	8 (20.5)	57 (32.2)
	Sub-total	108 (78.3)	20 (51.3)	128 (72.3)
Inpatient	Ward	16 (11.6)	9 (23.1)	25 (14.1)
	ICU	14 (10.1)	10 (25.6)	24 (13.6)
	Sub-total	30 (21.7)	19 (48.7)	49 (27.7)
	Total	138	39	177

*ICU* Intensive care unit

### Antibiotic resistance patterns of the isolates

The lowest rate of resistance was seen toward imipenem followed by piperacilli/tazobactam, amikacin and cefoperazone/sulbactam. All isolates were found to be resistant to amoxicillin (Table 3). Out of total 177 isolates, 96 (54.2%) were multidrug resistant. Among 138 *E. coli* and 39 *K. pneumoniae*, 73 (52.9%) *E. coli* and 23 (59%) *K. pneumoniae* were multidrug resistant.

**Table 3:** Antibiotic resistance patterns of *E. coli* and *K. pneumoniae* isolated

Antibiotics used	Resistance patterns of the isolates		Total (%)
	*E. coli* (N = 138) (%)	*K. pneumoniae* (N = 39) (%)	
Amikacin	10 (7.2)	9 (23.1)	19 (10.7)
Amoxicillin	138 (100)	39 (100)	177 (100)
Cefalexin	124 (89.8)	37 (94.9)	161 (91)
Ceftriaxone	117 (84.8)	33 (84.6)	150 (84.7)
Cefotaxime	120 (87)	33 (84.6)	153 (86.4)
Ceftazidime	114 (82.6)	33 (84.6)	147 (83)
Cefoperazone/sulbactam	12 (8.7)	8 (20.5)	20 (11.3)
Imipenem	3 (2.2)	6 (15.4)	9 (5.1)
Ciprofloxacin	68 (49.3)	18 (46.2)	86 (48.6)
Ofloxacin	68 (49.3)	18 (46.2)	86 (48.6)
Piperacillin/tazobactam	9 (6.5)	10 (25.6)	19 (10.7)
Co-trimoxazole	75 (54.3)	20 (51.3)	95 (53.7)

### ESBL and MBL production among the isolates

Out of 177 isolates, 121 isolates of *E. coli* and 33 isolates of *K. pneumoniae* were suspected as ESBL producers on primary screening test. Among them, 46 isolates of *E. coli* and 15 isolates of *K. pneumoniae* were confirmed as ESBL producers. Of the total isolates 61 (34.5%) were ESBL producers. Of total 61 ESBL positive strains detected, 51 were detected by both ceftazidime and ceftazidime/clavulanic acid discs and cefotaxime and cefotaxime/clavulanic acid discs, while 4 were detected by ceftazidime and ceftazidime/clavulanic acid discs only and 6 by cefotaxime and cefotaxime/clavulanic acid discs only.

Similarly, 9 isolates (3 *E. coli* and 6 *K. pneumoniae*) were suspected as MBL producers on the basis of resistance to imipenem. 7 (2 *E. coli* and 5 *K. pneumoniae*) (4%) isolates were found to be MBL producers.

### Distribution of ESBL and MBL producing isolates on the basis of samples

The distribution of ESBL producing strains according to different samples is presented in Table 4. Similarly, 1 of MBL producing bacterium was isolated from each urine, catheter tip and pus samples, while 2 MBL producing bacteria were isolated from each sputum and suction tip.

**Table 4:** Distribution of ESBL producing isolates on the basis of samples

Samples	Overall ESBL (%)	ESBL (%)	
		*E. coli* (n = 46)	*K. pneumoniae* (n = 15)
Urine	43 (70.5)	38 (82.6)	5 (33.3)
Catheter tips	3 (4.9)	0 (0)	3 (20.0)
Sputum	4 (6.6)	0 (0)	4 (26.7)
ET suction tips	0 (0)	0 (0)	0 (0)
Pus	7 (11.5)	6 (13.0)	1 (6.7)
Blood	3 (4.9)	1 (2.2)	2 (13.3)
Wound swab	1 (1.6)	1 (2.2)	0 (0)
Total	61 (100)	46 (100)	15 (100)

### Distribution of ESBL and MBL producing bacteria on the basis of type and department of patients

Most of the ESBL producing isolates were from outpatients. Among the outpatients, most of the isolates were from general medicine (43.3%) followed by obstetrics and gynecology department (27.9%). Similarly, among the inpatients, ESBL producing bacteria were more frequent in intensive care unit (16.4%) than wards (8.2%) (Table 5). MBL producing strains were not isolated from outpatients. 3 MBL producing bacteria were isolated from intensive care unit and 4 from wards.

**Table 5:** Distribution of ESBL producing bacteria on the basis of type and department of patients

Patient types	Departments of patient	*E. coli* (%)	*K. pneumoniae* (%)	Total (%)
Outpatient	Emergency	2 (4.3)	0 (0)	2 (3.3)
	Obstetrics and gynecology	15 (32.6)	2 (13.3)	17 (27.9)
	Pediatric	2 (4.3)	1 (6.7)	3 (4.9)
	General medicine	20 (43.5)	4 (26.7)	24 (39.3)
	Sub-total	39 (84.8)	7 (46.7)	46 (75.4)
Inpatient	ICU	5 (10.9)	5 (33.3)	10 (16.4)
	Ward	2 (4.3)	3 (20.0)	5 (8.2)
	Sub-total	7 (15.2)	8 (53.3)	15 (24.6)
	Total	46 (100)	15 (100)	61 (100)

### Resistance patterns of ESBL and MBL producing isolates

No ESBL producing strains were found to be resistant to imipenem. Similarly, among other antibiotics tested lowest rate of resistance was seen toward cefoperazone/sulbactam, piperacillin/tazobactam and amikacin (Table 6). MBL producing strains showed high rates of resistance to the antibiotics tested.

**Table 6:** Resistance patterns of ESBL producing isolates

Antibiotics	*E. coli* (%)	*K. pneumoniae* (%)	Total (%)
Co-trimoxazole	28 (60.9)	8 (53.3)	36 (59)
Cephalexin	46 (100)	15 (100)	61 (100)
Amoxicillin	46 (100)	15 (100)	61 (100)
Amikacin	3 (6.5)	2 (13.3)	5 (8.2)
Ciprofloxacin	28 (60.9)	8 (53.3)	36 (59)
Ofloxacin	28 (60.9)	8 (53.3)	36 (59)
Ceftriaxone	40 (87)	13 (86.7)	53 (86.9)
Ceftazidime	43 (93.5)	15 (100)	58 (95.1)
Cefotaxime	45 (97.8)	15 (100)	60 (98.4)
Cefoperazone/sulbactam	1 (2.2)	2 (13.3)	3 (4.9)
Pipercillin/tazobactam	2 (4.3)	2 (13.3)	4 (6.6)
Imipenem	0 (0)	0 (0)	0 (0)

## Discussion

Similar rate of growth positivity as in our study was also reported by Poudyal et al. (16.9%) [17]. In our study, *E. coli* (51.5%) and *K. pneumoniae* (14.7%) were most frequently isolated gram negative bacteria. Similar isolation rates for *E. coli* and *K. pneumoniae* were also reported by Poudyal et al. [17]. *E. coli* and *K pneumoniae* are among the commonest bacteria isolated from clinical specimens. Majority of the isolates in our study were from urine samples. This may be due to the larger number of urine samples included in our study. Further, *E. coli* and *K. pneumoniae* are common cause of urinary tract infection [18].

Higher numbers of isolates were from outpatient department in comparison to inpatient department. This may be attributed to the larger number of samples being included from outpatients.

Misuse of antibiotic is responsible for higher incidence of antibiotic resistance among bacteria [19]. We found the incidence of multidrug resistant bacteria to be 54.2%, with 52.9% of *E. coli* and 59% of *K. pneumoniae* being multidrug resistant. Different studies in Nepal have found the rates of multidrug resistance among *E. coli* to be ranging from 38.2 to 95.52% and those for *K. pneumoniae* to be 25–100% [17, 20, 21]. Common risk factors associated with infection by multidrug resistant bacteria are hospitalization and previous use of antibiotics [22].

In our study the rate of ESBL production was 34.5 with 33.3% of the *E. coli* and 38.5% of the *K. pneumoniae* being ESBL positive. The prevalence of ESBL producing *E. coli* and *K. pneumoniae* was found as low as 18.2 and 4.1% respectively in a study conducted by Raut et al. [23] and as high as 80% for *E. coli* [17] and 90.9% for *K. pneumoniae* [24]. The worldwide prevalence of ESBL production among the clinical isolates is found to be ranging from < 1 to 74% [25].

In our study, cefotaxime-clavulante combination disc identified numerically more confirmed ESBL producers in comparison to ceftazidime-clavulanate, which was analogous to the findings by Poudyal et al. [17] and Ranjini et al. [26].

Lower rate of MBL production in comparison to the study by Bora et al. (*E. coli* = 18.98%, *K. pneumoniae* = 21.08%) was reported in our study [9]. However, in other studies conducted in different countries showed the rates of MBL production to be ranging from 13.4 to 61.5% for *E. coli* and 33–36% for *K. pneumoniae* [27–29].

In our study high rate of ESBL production was observed among the bacteria isolated from out patients, which is very serious and shows the dissemination of ESBL producing bacteria to the community. However, no MBL producing organism was isolated from outpatients.

The prevalence of drug resistant bacteria may not only vary from countries to countries but also from institutions to institutions and this can be partially explained by the difference in local antibiotic prescribing habits and difference in effectiveness of infection control program in different health institutes.

In our study, the highest rate of susceptibility of the bacteria was found toward imipenem followed by pipercillin/tazobactum, amikacin and cefoperazone/sulbactum. These findings were in harmony with the findings of other studies conducted by Ansari et al. [30], Kader and Kumar [31] and Shashwati et al. [32].

## Conclusions

ESBL and MBL production along with multidrug resistance among *E. coli* and *K. pneumoniae* are presenting as the serious problem in Nepal. The high rate of ESBL production among the isolates from outpatients is very serious issue, which suggests the dissemination of ESBL producing isolates in community. Regular monitoring of rate of ESBL and MBL production along with multidrug resistance among these clinical isolates is very necessary. Further, to control the emergence of drug resistance strict policy to rationalize the use of antibiotics is necessary. On the basis of the drug resistance patterns we found in our study imipenem followed by piperacilli/tazobactam, amikacin and cefoperazone/sulbactam may be used for the preliminary treatment of the infections caused by *E. coli* and *K. pneumoniae* in our setting.

## Abbreviations

ESBL: extended spectrum beta-lactamase
MBL: metallo beta-lactamase
MDR: multidrug resistant
ICU: intensive care unit
